# The Interactive Effects of Post-Traumatic Stress Symptoms and Breathlessness on Fatigue Severity in Post-COVID-19 Syndrome

**DOI:** 10.3390/jcm11206214

**Published:** 2022-10-21

**Authors:** Sari Harenwall, Suzanne Heywood-Everett, Rebecca Henderson, Joanne Smith, Rachel McEnery, Amy R. Bland

**Affiliations:** 1 Primary Care Wellbeing Service, Bradford District Care NHS Foundation Trust, Shipley BD18 3LD, UK; 2 Department of Psychology, Manchester Metropolitan University, Manchester M15 6BH, UK

**Keywords:** long-COVID, fatigue, PTSD, breathlessness, rehabilitation, post-COVID syndrome, dyspnoea

## Abstract

Background: Post-traumatic stress symptoms (PTSS) and breathlessness have been well documented in the acute phase of COVID-19 as well as in Post-COVID-19 Syndrome (PCS), commonly known as Long-COVID. The present study aimed to explore whether PTSS and breathlessness interact to exacerbate fatigue among individuals recovering from PCS, similar to the effects evidenced in other health conditions that feature respiratory distress.. Methods: Outcome measures were collected from 154 participants reporting persistent fatigue following acute COVID-19 infection who were enrolled in a 7-week rehabilitation course provided by the Primary Care Wellbeing Service (PCWBS) in Bradford District Care NHS Foundation Trust (BDCFT). Results: Hierarchical multiple linear regression revealed that fatigue severity was associated with a significant interaction between PTSS and breathlessness, even when controlling for pre-COVID health related quality of life (HRQoL), age, symptom duration and hospital admittance during the acute phase. Furthermore, improvements in fatigue following rehabilitation were significantly associated with improvements in PTSS. Conclusions: PTSS may be an important therapeutic target in multidisciplinary rehabilitation for reducing fatigue in the recovery from PCS. It is therefore important that treatment for PCS takes a biopsychosocial approach to recovery, putting emphasis on direct and indirect psychological factors which may facilitate or disrupt physical recovery. This highlights the need for all PCS clinics to screen for PTSD and if present, target as a priority in treatment to maximise the potential for successful rehabilitation.

## 1. Introduction

It is now well-established that Coronavirus disease 2019 (COVID-19), caused by severe acute respiratory syndrome coronavirus 2 (SARS-CoV-2), can result in persistent symptoms lasting beyond the resolution of the acute infection [1]. Persistent symptoms lasting for more than 12 weeks have been characterised as post-COVID-19 syndrome (PCS), also commonly referred to as “long-COVID”. It is currently estimated that 10–35% of people recovering from acute COVID-19 infection present with PCS [2]. Symptoms of PCS are both physical and psychological [3] with frequently reported symptoms including fatigue, breathing difficulties, anxiety, depression, cognitive dysfunction and post-traumatic stress [4,5]. Persistent physical and psychological symptoms following acute COVID-19 infection significantly impact quality of life, capacity to work, and performance of usual daily activities [6]. Moreover, studies estimate that only a quarter of people with PCS have resumed their usual working activity [4].

Fatigue is the most frequently reported symptom in PCS, and is the most persistent symptom at 6-months follow up [4]. Likewise, post-viral fatigue syndrome and myalgic encephalomyelitis(ME)/Chronic Fatigue Syndrome (CFS) have been observed previously in similar viral outbreaks such as SARS in Hong Kong in 2003 which showed subsequent clusters that were present even at a 4-year follow-up [7]. A systematic review of biological, psychological, and social predictors of post-viral fatigue syndrome revealed that biological markers of the severity of the acute infection were the most consistent predictors, with some support for self-reported anxiety, perceived stress, negative beliefs about the acute illness, and premorbid distress [8]. Indeed, it is widely recognised that both biological and psychological conditions may manifest with fatigue, or co-occur with a post-viral fatigue state [9].

Previous research has suggested a relationship between Post-Traumatic Stress Disorder (PTSD) and fatigue, specifically ME/CFS [10] and fatigue in population-based samples [11]. According to DSM-5 criteria, PTSD includes witnessing a traumatic event, persistently re-experiencing that event (intrusive memories, nightmares), avoiding thoughts and feelings about the trauma, and changes in negative mood and arousal which cause significant distress and functional impairment. Importantly, PTSD can arises following exposure to traumatic events perceived to represent a threat to life, including life-threatening illnesses [12] such as COVID-19. Whilst the exact underlying mechanisms are unknown, the hypothalamo–pituitary–adrenal (HPA) axis plays a major role in regulating stress responses [13,14]. Likewise, changes in the HPA axis are hallmarks of PTSD [15], ME/CFS [16] and post-viral fatigue [17]. It is likely that, similarly to ME/CFS, fatigue in PCS may be exacerbated by PTSD. Indeed, evidence is now emerging to suggest that PTSD is consistently identified in those with living with PCS [18]. Likewise, previous epidemics have shown an increased risk of PTSD among survivors following recovery from both SARS and MERS [19,20]. PTSD may be higher among those who experienced severe illness due to COVID-19, especially those admitted to an intensive care unit (ICU) or hospitalised with severe illness [21]. However, PTSD has also been widely reported in the general population due the psychosocial impact of social isolation, uncertainty of disease progression, financial losses, increased perception of risk, etc. [22].

It is estimated that 17–44% of those recovering from critical illness experience PTSD [23] and a recent systematic review of the neuropsychiatric sequelae of COVID-19 found PTSD were reported in a third of studies, with between 6.5% and 42.8% of participants affected [24]. PTSD presents with a range of symptoms such as flashbacks, nightmares, hyperarousal, insomnia, avoidance, and cognitive and functional impairments [25]. Nevertheless, many people who experience post-traumatic stress symptoms (PTSS) may not reach the DSM-V criteria for PTSD. Indeed, even among those who do not meet full diagnostic criteria, PTSS have also been associated with functional impairment and PTSD may require a differential diagnostic consideration for COVID-19 survivors [21]. This highlights the need to explore sub-clinical levels of post-traumatic stress amongst people living with post-acute COVID-19 sequelae.

Heightened death anxiety has been widely reported in relation to the COVID-19 pandemic and is associated with psychological distress and an increased perception of the threat posed by the virus [26]. Therefore, it is likely that infection with SARS-CoV-2 can be perceived as a life-threatening event, increasing the risk of PTSS, particularly among those considered most at-risk [27]. Findings from Horn et al. [28] and Matalon et al. [29] support this hypothesis, with psychological distress at the onset of COVID-19 illness predictive of subsequent PTSS. However, PTSS have also been reported among patients who experienced a mild initial illness with COVID-19 and did not require hospitalisation. For example, Ismael et al. [30] investigated post-infection PTSS among mild COVID-19 cases in Brazil. They found a clinically significant level of PTSS was reported by 17.3% of participants, with more COVID-19 symptoms in the acute stage of the illness increasing the risk of post-infection PTSS. These findings are supported elsewhere, with symptom load found to predict PTSS among both hospitalised and non-hospitalised COVID-19 survivors in Norway [31].

Breathlessness during the acute infection has also been identified as a risk factor for PTSS symptoms in both hospitalised and non-hospitalised individuals recovering from COVID-19 [31], with researchers suggesting that symptoms such as breathlessness increase threat perceptions and cause significant distress [32]. Breathlessness is defined as a subjective experience of breathing discomfort that arises due to an interplay between physiological, psychological, social, and environmental factors [33]. Indeed, dyspnea is a potentially psychologically traumatising sensation [34] which is associated with neural activation of the emotional salience network, including the anterior insula, anterior cingulate cortex, and amygdala [35]. Furthermore, people with PTSD, compared with those without, have been shown to have a significantly increased risk for airflow limitation [36]. Similarly to PTSS, chronic breathlessness causes excessive stimulation of the HPA axis, resulting in dysfunctional regulation of the HPA axis [37]. Taken together, it is possible that breathlessness and PTSS may interact to exacerbate outcomes in people with PCS, with such interactions observed in other health conditions that feature breathlessness and respiratory distress. For example, both breathlessness and psychological distress have been associated with fatigue severity in chronic obstructive pulmonary disease (COPD [38]), and myocardial infarction [39]. Additionally, research suggests that in COPD, breathlessness may be perceived as a near-death experience, with the prevalence of PTSS increasing as the individual experiences further COPD exacerbations [40]. These findings suggest that fatigue may be, at least in part, exacerbated by increased psychological distress arising because of physical symptoms, such as breathlessness. Consequently, although research has not yet adequately considered the potential associations between breathlessness, PTSS and fatigue in PCS, it seems plausible that breathlessness and PTSS may interact to exacerbate fatigue among individuals recovering from PCS, similar to the effects evidenced in other health conditions that feature respiratory distress, such as COPD and asthma.

The present study therefore sought to test this hypothesis, exploring whether PTSS symptoms and breathlessness are related to fatigue severity in a sample of patients presenting with PCS fatigue, and whether improvements to PTSS and breathlessness would therefore predict improvements to fatigue scores following multidisciplinary rehabilitation.

## 2. Materials and Methods

### 2.1. Participants

Participants were enrolled on a 7-week rehabilitation course specifically targeting Health Care Professionals (HCPs) with persistent symptoms of COVID-19, offered by the Primary Care Wellbeing Service (PCWBS) in Bradford District Care NHS Foundation Trust (BDCFT) [41]. A total of 154 participants completed outcome measures detailing their pre-COVID-19 and current state of health. The sample comprised 87% female and 13% male participants, with a mean age of 47.29 years (range: 22–68, Standard Deviation (S.D).10.44). 73% identified as White British, 17% as South Asian (Pakistani, Indian, Bangladeshi, British Asian) and 10% as other ethnicities. All participants lived and/or worked in the Bradford, Craven and Airedale district, with 76% of participants working as social, health and care staff across the district, including trust, council and care home staff. Following the 7-week multidisciplinary rehabilitation course provided by the PCWBS, 79 participants completed post-course outcome measures. Informed consent was obtained from all participants involved in the study.

### 2.2. Outcome Measures

Participants completed an adapted version of the C19-YRS (Yorkshire Rehabilitation Scale [42]) detailing specific symptoms related to PCS (Cronbach’s α = 0.891). As part of the questionnaire, participants were asked to report whether they had tested positive for COVID-19 during the acute phase, had been hospitalised and/or ventilated, and for how many days. Participants were also asked to rate their level of breathlessness (scale 0–10) with zero being no breathlessness and 10 being extreme breathlessness, fatigue (scale 0–10) with zero being no fatigue and 10 being extreme fatigue. Post-traumatic stress symptoms were measured using a 3-item scale developed as part of the C19-YRS [42]. Participants were asked to rate their level of PTSS on a scale from 0 = none, 1 = mild, 2 = moderate, 3 = severe and 4 = extreme. Participants were asked “(1) Have you had any unwanted memories of your illness or hospital admission whilst you were awake, not counting dreams?”, “(2) Have you had any unpleasant dreams about your illness or hospital admission?” and “(3) Have you tried to avoid thoughts or feelings about your illness or hospital admission”? Participants completed the EuroQol EQ-5D-5L, which assessed health-related quality of life (HRQoL) across five dimensions, including mobility, self-care, usual activities, pain/discomfort and anxiety/depression [43]. Each dimension is scored from 1 (no problems) to 5 (extreme problems). The scores from all five dimensions were combined and scaled using the UK standardised valuation study protocol (EQ-VT) value set, developed by the EuroQol group. EQ5D-5L indices were measured on a scale of 0 to 1, with 1 being perfect health and 0 being worst health, to provide an index that represents overall health-related quality of life (HRQoL). Participants also retrospectively rated their pre-COVID-19 HRQoL in order to control for potential confounding effects of their health status prior to SARS-CoV-2 infection.

### 2.3. Statistical Analysis

Statistical analyses were conducted in SPSS 27 and JASP (JASP Team (2020), version 0.14.1). We encountered ~5% missing data across the outcome variables and obtained estimates in the presence of missing scale data using the expectation maximization (EM) algorithm [44]. In order to examine whether PTSS and breathlessness are associated with fatigue, we used a combination of Frequentist and Bayesian hierarchical regressions. As opposed to frequentist statistics, Bayesian statistics does not compute *p* values but instead Bayes factors (typically, a Bayes factor of 3 corresponds to a *p*-value of 0.05 [45]). Taking a Bayesian perspective allowed us to further take a data-driven approach by exploring which model is the best model in terms of possible combinations of candidate predictor symptoms [46]. For the Bayesian analyses, the model prior was set to Uniform (i.e., all models are a priori equally likely) with Bayesian Adaptive Sampling (BAS) and the prior on parameters was set to Jeffreys-Zellner-Siow (JZS) prior (0.354). In both frequentist and Bayesian approaches, we included age, symptom duration, hospital admittance and pre-COVID HRQoL as covariates. In the next step, we included PTSS and breathlessness to observe main effects, and in the final step included a PTSS*breathlessness interaction term. Pearson’s r correlation coefficients showed that PTSS (unwanted memories, unpleasant dreams and thought avoidance) were highly correlated (all *p* < 0.0001) and therefore were combined to generate one PTSS variable. To evaluate symptom rating change between pre-course and post-course health, a series of repeated measures analysis of variance (ANOVA) were conducted for each symptom. The statistical significance level was set to *p* < 0.05 (two-tailed).

## 3. Results

### 3.1. Pre-COVID HRQoL

Participants (*N* = 154) reported a generally good level of health prior to their COVID-19 infection, with 10 participants indicating a long-term health condition (*n* = 1 COPD, *n* = 1 Lupus, *n* = 2 mobility problems, *n* = 1 hearing impairment, *n* = 2 diabetes, 3 = prefer not to say). The majority of participants reported none or slight health problems in the ED-5D-5L with a mean index of 0.88 (S.D. 0.18).

### 3.2. Post-COVID-19 Syndrome Symptom Impact on Fatigue Severity

All 154 participants reported problems with fatigue with a mean rating of 7.04 (S.D.2.18), 94% experienced breathlessness with a mean rating of 3.85 (S.D.2.64), and 61% of participants experienced PTSS with a mean rating of 1.29 (S.D.0.80). ED-5D-5L index scores fell to 0.54 (S.D.0.24).

37.7% of participants reported working as usual, whilst 21.3% were on a phased return, and 41.1% were on long-term sickness leave. 71% of participants had a positive COVID-19 test during the acute phase, however, widespread testing was not available at the start of data collection for the present study. 14% were admitted to hospital for an average of 11.62 days (S.D.17.79). The average duration of persistent symptoms was 6.02 months (range = 1–18; S.D.3.96). Of hospitalised patients, 91.3% of patients reported PTSS compared to 55.6% of non-hospitalised participants.

A hierarchical multiple regression analysis (Table 1), entering pre-COVID HRQoL, age, symptom duration and hospital admittance into the first step produced a significant model [*F* (4,150) = 2.90, *p* = 0.024] and accounted for 7% variance in fatigue (R^2^ = 0.07). Adding main symptom ratings for PTSS and breathlessness into step two explained an additional 8% of the variance [*F* (6,148) = 4.25, *p* = 0.001, R^2^ = 0.147], showing that breathlessness significantly predicted fatigue. However, PTSS did not, although there was a trend towards significance. The Bayesian regression analysis showed that the model with the highest Bayes Factor (BF_M_ = 15.04) included pre-COVID HRQoL, hospital admittance, breathlessness and PTSS.

**Figure 1:**
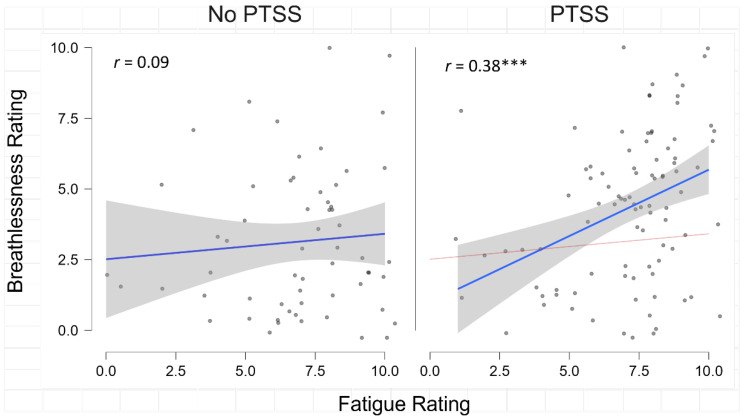
Correlations between fatigue and breathlessness in Post COVID-19 Syndrome (PCS) displayed separately by Post Traumtic Stress Symptoms (PTSS) group. *** *p* < 0.001.

In order to test for the interactive effects of PTSS and breathlessness, an interaction term was calculated. PTSS*breathlessness significantly improved the final model, which explaining 18% of the variance of fatigue, and was a significant predictor [β = 0.41, *t*  = 2.45, *p* = 0.016, *BF*_10_ = 20.97] causing the main effect of breathlessness to become non-significant (*p* = 0.633). Likewise, the Bayesian regression analysis showed that the model with the highest Bayes Factor (*BF*_M_ = 56.64) included pre-COVID HRQoL, hospital admittance, and the interaction of PTSS*breathlessness, suggesting that the interaction of PTSS and breathlessness is an important factor contributing to fatigue severity. Indeed, we observed a significant correlation between breathlessness and fatigue in participants who reported PTSS (*r* = 0.38, *p* < 0.001) but not in participants who were not experiencing PTSS (*r* = 0.09, *p* = 0.491) (Figure 1), suggesting that PTSS may exacerbate physical symptom presentation.

### 3.3. Post-Course Symptom Improvement

Given that pre-COVID HRQoL and hospital admittance were significant predictors of fatigue, we entered these as covariates in the repeated measures ANOVA to test for significant improvements in symptoms following rehabilitation (see Table 2). There was a statistically significant improvement in HRQoL [*F* (1,76) = 5.14, *p* = 0.026, *η_p_*^2^ = 0.06] fatigue [*F* (1,78) = 10.92, *p* = 0.001, *η_p_*^2^ = 0.12], and PTSS [*F* (1,79) = 4.01, *p* = 0.049, *η_p_*^2^ = 0.05]. Breathlessness showed a significant improvement; however this did not reach significance when accounting for pre-COVID HRQoL and hospital admittance [*F* (1,79) = 0.86, *p* = 0.771, *η_p_*^2^ = 0.01].

Correlational analyses revealed a significant association between improvements in fatigue and PTSS (*r* = 0.258, *p* = 0.023), but not fatigue and breathlessness (*r* = 0.122, *p* = 0.296). Changes in fatigue and PTSS were both significantly associated with improvements in HRQoL (*r* = −0.371, *p* < 0.001; *r* = −0.411, *p* < 0.001) but breathlessness was not (*r* = −0.170, *p* = 0.143). Regression analyses revealed that only pre-COVID HRQoL and PTSS symptom improvement significantly predicted fatigue improvement (Table 3). Bayesian analysis showed that the model with the highest Bayes factor (*BF*_M_ = 15.86) included pre-COVID HRQoL, hospital admittance and PTSS suggesting that PTSS improvement may be an important therapeutic target for reducing fatigue in PCS.

## 4. Discussion

The present study aimed to explore whether PTSS and breathlessness impact fatigue severity in PCS. Our results show that PTSS alone does not impact fatigue severity, but that PTSS significantly interacts with breathlessness to predict fatigue severity in PCS, even when controlling for pre-COVID HRQoL, age, symptom duration and hospital admittance. Furthermore, following the 7-week rehabilitation “Recovering from COVID” course, improvements in PTSS were significantly associated with improvements in fatigue suggesting that PTSS may be an important therapeutic target for multidisciplinary rehabilitation.

The aetiology of PTSS among those with PCS is complex and multifaceted, potentially arising from both the infection itself and as an outcome of the wider psychosocial context associated with the COVID-19 pandemic [22]. Given that the majority of participants in the present study were health care professionals (HCPs), they are likely to be at particular risk of developing PTSS. Previous research suggests an estimated 45% of front line HCP reporting persistent symptoms of COVID-19 and 32% struggling to cope [47]. HCPs face enormous pressure, including a high risk of infection, inadequate protection from contamination, overwork/longer-shifts, isolation, a lack of contact with their families, and exhaustion [48,49,50]. HCPs have been shown to be at greater risk of myalgias (fatigue, muscle soreness) compared to the general population [51]. Not only are HCPs at an increased risk of the physical consequences of exposure to COVID-19 infection, they have also been exposed to high levels of stressful or traumatic events which have been shown to have substantial negative mental health outcomes [52]. A relatively high prevalence of anxiety (25%), depression (25%) and sleep disorders (44%) has been reported in meta-analyses investigating the mental health of HCPs [53,54]. However, despite the challenges in differentiating between the various potential causes of PTSS, there is still a pressing need to consider the impact such psychological comorbidities may have on physical recovery from COVID-19, particularly among those with PCS. Although it has been established that PTSS are prevalent in those suffering from PCS, little research has examined the impact of these symptoms on rehabilitation and recovery. Our results suggests that PTSS is an important factor which may influence both fatigue severity and fatigue reduction following PCS rehabilitation.

Our findings are also in line with previous literature that suggests a link between PTSD and respiratory dysfunction [55,56]. Whilst the exact underlying mechanisms are unknown, evidence suggests that this relationship may be mediated by inflammatory processes leading to a pro-inflammatory state [36]. This, in combination of the respiratory dysfunction experienced during both the acute phase of COVID-19 and the persistent sequelae, is likely to lead to cyclical breathing difficulties which may further exacerbate PTSS and, thus, fatigue. Bower et al. [57] argue that both infection with SARS-CoV-2 and stressors encountered as a consequence of the pandemic may contribute to fatigue and psychiatric symptoms through overactivation of the proinflammatory cytokine network. They propose that distressing psychological states may increase inflammation, with an association between fatigue and elevated inflammatory markers noted in other health conditions. However, findings in PCS are mixed, with one study finding no association between fatigue and inflammatory markers in PCS [58]. Nevertheless, psychoneuroimmunology research suggests that reducing distress may reduce such inflammation [59]. Indeed, our results support the concept that reducing distress, i.e., PTSS, is related to reduced fatigue, a core physical manifestation of PCS.

Previous literature has also shown well established links between breathlessness and fatigue severity [60]. However, our results demonstrated that breathlessness was no longer a significant predictor of fatigue when the interaction of PTSS and breathlessness was added into the regression model. This suggests that breathlessness alone may not significantly impact fatigue severity in PCS. Indeed, although damage to the respiratory system in some individuals recovering from COVID-19 has been demonstrated [61], all participants included in the present study showed no permanent lung damage during x-ray investigations. Consequently, this reported breathlessness may be a result of breathing pattern disorders (BPD) which are a common cause of chronic breathlessness, including after acute respiratory illnesses such as COVID-19 [62]. However, further research is needed to establish whether potential BPDs pre-date acute COVID (and hence presents as a vulnerability for both acute infection and PCS), or whether it is a result of people adopting a dysfunctional breathing pattern during the acute phase of COVID due to restricted airflow and blocked sinuses that continues beyond the acute phase. 

It is also important to note that the biological mechanisms underpinning breathlessness extend beyond this organ system. Indeed, breathlessness is defined as a subjective experience, with physiological and affective manifestations [33]. Distinct neuronal pathways have been demonstrated for these manifestations, with affective dimensions of breathlessness associated with the limbic system, whereby information from vagal afferents in the lungs projects to the amygdala, medial dorsal areas of the thalamus and the insula [63]. Importantly, the limbic system is also associated with PTSS [64], with evidence to suggest that interactions between breathlessness and PTSS may manifest in this emotional neural network. For example, breathlessness has been found to heighten amygdala responses during affective processing [65], while negative affect has simultaneously been found to increase breathlessness perception [66]. These findings suggest that there may be a bidirectional link between the negative affect arising due to PTSS and breathlessness. Specifically, PTSS may intensify perceptions of breathlessness in those with PCS, while breathlessness may simultaneously heighten the negative affect arising due to PTSS. This is particularly relevant when considering that the amygdala is thought to regulate activation of the HPA axis [67], with HPA dysregulation a core feature in ME/CFS [68]. Although this has not been established as a causal mechanism, and may arise as a consequence of fatigue, it is plausible that the psychological distress associated with PTSS and breathlessness may contribute to fatigue through the overactivation of the amygdala and subsequent HPA dysregulation. This is an important avenue for future research that seeks to clarify the biological mechanisms of fatigue in PCS.

Taken together, it is important that treatment for PCS takes a biopsychosocial approach to recovery, putting emphasis on direct and indirect psychological factors which may facilitate or disrupt physical recovery such as the impact on systemic inflammation and coping behaviours (including engagement in rehabilitation). Importantly, there is also evidence to suggest that the presence of mental health comorbidities, such as PTSS, may reduce an individual’s capacity to engage in self-management behaviours for other health conditions (e.g., diabetes [69]). Since self-management is central to current post-COVID-19 syndrome rehabilitation guidelines [25], it is likely that increased PTSS may impede individuals’ ability to engage in behaviours that may support recovery from PCS. Furthermore, as some of the core presentation of PTSS involves avoidance of difficult thoughts, memories, feelings, and sensations, it is possible that for some people, the very nature of a rehabilitation program can become another trigger which contributes to the maintenance or even exacerbation of PCS, should PTSS be neglected—although further research is required to test this assumption. Evidence from randomised clinical trials suggest that people are unlikely to recover from PTS disorders without formal treatment [70], [71] therefore underscoring the need to address PTSS as a preventative strategy to ameliorate PTSD in PCS.

### Limitations

The present study has several limitations. Firstly, the sample of participants had self-referred onto the “recovering from COVID” course and were seeking help for PCS, particularly regarding fatigue. These results may therefore not be generalisable to a wider sample of PCS exhibiting an array of post COVID-19 sequalae. Likewise, the majority of participants were HCPs and were therefore more likely to have experienced traumatic events. Second, response rates for outcome measures were low with only half of participants completing the post-course assessments. This may cause potential bias regarding participants who completed the responses and those that did not. Third, the outcome measures used for PTSS and fatigue were taken from a COVID specific outcome measurement and further research into the nuances of specific PTSS and fatigue should be generated with wider use of validated questionnaires.

## 5. Conclusions

- In conclusion, it is possible that improving mental health comorbidities associated with PCS may not only improve mental health and quality of life among COVID-19 survivors, but may also help to reduce some of the physical symptoms associated with PCS.
- The present study demonstrates that interactions between breathlessness and PTSS may contribute to the persistent fatigue observed in PCS and that improvements in PTSS may lead to improvements in reported fatigue. This suggests that PTSS may be an important therapeutic target for multidisciplinary rehabilitation in recovery from PCS.
- The results from the present study highlight the need to move beyond the dichotomy of mental and physical heath and move towards a framework where we consider the interplay between mental and physical health. The treatment models for PCS currently being developed nationally are in a unique position to progress this further without the traditional barriers of commissioning, which often separate the two.

### 5.1. Clinical Implementations

Encouragingly, PTSS can be easily screened for in the clinic with a number of effective evidence-based treatments for PTSD (e.g., NICE guidance for PTSD [72]), where often dysfunctional breathing, nightmares and avoidance are key therapeutic targets. As such, it is important for all PCS clinics to screen for PTSS and if present, target as a priority in treatment alongside other contributing factors to maximise the potential for successful rehabilitation within an integrated, multidisciplinary biopsychosocial framework.

### 5.2. Future Directions

There are a number of research priorities highlighted from these findings:

- It will be important to investigate to what extent PTSS is a barrier to effective PCS rehabilitation, including self-help, group and individual treatment to develop more helpful clinical guidelines and to establish the cut-off for PTSS in PCS.
- More emphasis is needed on understanding patient characteristics from a biopsychosocial perspective, in order to better triage and assign to effective treatment modalities. Ideally, this should move beyond symptoms and the absence of symptoms, and include meaningful measures of function and coping amongst people living with PCS.
- Finding cost effective treatment for PCS needs to be a priority for a number of reasons; including the disproportionally higher prevalence of PCS in HCP’s, putting healthcare services under further strain due to high levels of sickness absence and further risk of burnout.

## Figures and Tables

**Table 1:** Summary of hierarchical multiple regression analyses for predictors of fatigue in PCS.

Model	Predictors	β	*t*	*p*	*BF*_10_
1	pre-COVID HRQoL	−0.23	−2.93	0.004 **	3.43
*R*^2^ = 0.07	age	−0.04	−0.54	0.593	0.39
	symptom duration	0.00	0.04	0.966	0.35
	hospital admittance	−0.15	−1.90	0.060	1.06
2	pre-COVID HRQoL	−0.23	−2.90	0.004 **	7.64
*R*^2^ = 0.147	age	−0.04	−0.46	0.647	0.82
	symptom duration	0.01	0.12	0.906	0.76
	hospital admittance	−0.21	−2.64	0.009 **	4.82
	PTSS	0.15	1.72	0.088	1.80
	breathlessness	0.20	2.51	0.013 *	11.39
3	pre-COVID HRQoL	−0.23	−2.99	0.003 **	21.60
*R*^2^ = 0.180	age	−0.01	−0.07	0.942	0.64
	symptom duration	0.03	0.36	0.716	0.65
	hospital admittance	−0.24	−2.96	0.004 **	16.96
	PTSS	−0.13	−0.95	0.346	0.99
	breathlessness	0.05	0.48	0.633	0.85
	PTSS * breathlessness	0.41	2.44	0.016 *	20.97

* *p* < 0.05, ** *p* < 0.01. PCS = Post COVID-19 Syndrome; HRQoL = Health Related Quality of Life; PTSS = Post Traumtic Stress Symptoms.

**Table 2:** Symptom ratings pre- and post-rehabilitation.

Outcome Measure	Pre-Course Rating	Post-Course Rating
HRQoL	0.55 (0.21)	0.61 (0.24) *
Fatigue (0–10)	7.08 (2.34)	6.47 (2.40) ***
PTSS (0–4)	0.72 (0.08)	0.68 (0.09) *
Breathlessness (0–10)	3.96 (2.88)	3.19 (2.25)

Note: conditioned on variables: Hospital admittance, Pre-COVID Health Related Quality of Life (HRQoL). * *p* < 0.05, ** *p* < 0.01, *** *p* < 0.001. PTSS = Post Traumtic Stress Symptoms.

**Table 3:** Summary of hierarchical multiple regression analyses for predictors of fatigue improvement in PCS following rehabilitation.

Model	Predictors	β	*t*	*p*	*BF*_10_
1	pre-COVID HRQoL	−0.33	−3.01	0.003 **	6.54
*R*^2^ = 0.12	hospital admittance	−0.21	−1.95	0.055	2.04
2	pre-COVID HRQoL	−0.40	−3,56	<0.001 ***	12.23
*R*^2^ = 0.19	hospital admittance	−0.21	−2.00	0.053	2.60
	PTSS change	0.24	2.12	0.037 *	3.57
	Breathlessness change	0.06	0.58	0.562	1.00
3	pre-COVID HRQoL	−0.39	−3.51	<0.001 ***	8.92
*R*^2^ = 0.20	hospital admittance	−0.20	−1.89	0.063	2.15
	PTSS change	0.23	2.06	0.043 *	2.55
	Breathlessness change	0.07	0.65	0.520	0.80
	PTSS*breathlessness change	−0.10	−0.91	0.364	0.85

* *p* < 0.05, ** *p* < 0.01, *** *p* < 0.001. PCS = Post COVID-19 Syndrome; HRQoL = Health Related Quality of Life; PTSS = Post Traumtic Stress Symptoms.

## Data Availability

Data is not openly available.
